# Assessment of a Diterpene-Rich Rosemary (*Rosmarinus officinalis* L.) Extract as a Natural Antioxidant for Salmon Pâté Formulated with Linseed

**DOI:** 10.3390/antiox11061057

**Published:** 2022-05-26

**Authors:** Cristina Cedeño-Pinos, Magdalena Martínez-Tomé, Dario Mercatante, María Teresa Rodríguez-Estrada, Sancho Bañón

**Affiliations:** 1 Department of Food Technology and Science and Nutrition, Veterinary Faculty, Regional Campus of International Excellence “Campus Mare Nostrum”, University of Murcia, 30100 Murcia, Spain; cristinacarmen.cedenop@um.es (C.C.-P.); mmtome@um.es (M.M.-T.); 2 CIBER: CB12/03/30038 Pathophysiology of Obesity and Nutrition, CIBERobn, Carlos III Health Institute (ISCIII), 28013 Madrid, Spain; 3 Department of Agricultural and Food Sciences, Alma Mater Studiorum-Università di Bologna, 40127 Bologna, Italy; dario.mercatante2@unibo.it (D.M.); maria.rodriguez@unibo.it (M.T.R.-E.)

**Keywords:** polyphenols, diterpenes, carnosic acid, carnosol, natural antioxidants, fish products

## Abstract

The use of natural plant extracts with standardised antioxidant properties is a growing strategy to stabilise food products. The use of a rosemary lipophilic extract (RLE), obtained from the by-product of high-yield selected plants and rich in polyphenols (334 mg/g, with diterpenes such as carnosic acid and carnosol as main compounds), is here proposed. Four RLE doses (0, 0.21, 0.42 and 0.63 g/kg) were tested in a salmon pâté formulated with sunflower oil and linseed, which was pasteurised (70 °C for 30 min) and subjected to storage at 4 °C and 600 lux for 42 days. Rosemary diterpenes resisted pasteurisation without degrading and showed antioxidant activities during the shelf-life of pasteurised pâté. RLE addition led to increased peroxide value (from 3.9 to 5.4 meq O_2/_kg), but inhibited formation of secondary oxidised lipids such as malondialdehyde (from 1.55 to 0.89 mg/g) and cholesterol oxidation products (from 286 to 102 µg/100 g) and avoided discolouration (slight brownness) in the refrigerated pâté. However, this did not entail relevant changes in fatty acid content or in the abundance of volatile organic compounds from oxidised lipids. Increasing the RLE dose only improved its antioxidant efficacy for some oxidation indexes. Thus, the oxidative deterioration of these types of fish emulsion can be naturally controlled with rosemary extracts rich in diterpenes.

## 1. Introduction

Fish emulsions, such as salmon pâté, represent a valuable dietary source of polyunsaturated fatty acids (PUFA), in particular, of some essential long-chain n-3 PUFA, such as eicosapentaenoic acid (EPA) and docosahexaenoic acid (DHA) [1]. The intake of these two PUFA has been correlated with a lesser risk of cardiovascular diseases [2], neurodegenerative diseases and inflammatory disorders [3]. However, PUFA are particularly prone to oxidation and can be degraded during manufacturing and storage of fish pâté due to oxidising factors such as oxygen, iron, salt, heating and mechanical treatments [4,5]. Lipid oxidation can generate secondary compounds, such as malondialdehyde (MDA) and cholesterol oxidation products (COP), together with volatile organic compounds (VOC), including hydrocarbons, aldehydes, ketones and alcohols, among others, responsible for flavour and rancidity [6]. An excessive intake of oxidised lipids may increase the risk of some pathologies such as metabolic and neurodegenerative diseases and some cancers [7,8]. Oxidation reactions involving other pâté components may also lead to some changes in colour and pH. Therefore, it is justified that fish pâté, a product rich in unsaturated fat, is stabilised with antioxidants. Synthetic antioxidants, including tertiary butylated hydroquinone, butylated hydroxy anisole, propyl gallate and butylated hydroxy toluene (BHT), are used in the food industry to stabilise food lipids due to their low cost and high efficiency [9]. Nevertheless, some of these antioxidants are being questioned due to their technological limitations (low dosage, thermal sensibility and high volatility) [10], and, in particular, to their possible role as toxicological and carcinogenic agents in humans [11,12].

Nowadays, “green label” formulations for fish products often include “generally recognised-as-safe” (GRAS) natural preservatives. Among these, phenolic compounds present in fruits, vegetables, beverages (tea, wine, juices), plants, seaweed and some herbs show antioxidant and antimicrobial activities, considered as emerging natural additives for fish products [13]. Phenolic compounds would mainly have an antioxidant role in fish products where microbiological quality is ensured by heating treatments. In recent studies, oxidative stability could be improved in different fish products (burgers, cooked and frozen cuts) using different phenolic extracts (rosemary, sage, lavender, moringa, pomegranate and olive) [14,15,16,17,18]. These studies reveal the importance of using active ingredients with suitable properties (e.g., solubility and antioxidant power) to obtain good results in fish matrixes.

Rosemary (*Rosmarinus officinalis* L.) is considered a source of phenolic antioxidants, being particularly rich in some hydrophobic diterpenes such as carnosic acid and carnosol, which can act as free radical scavengers in lipid matrixes [19]. The rosemary plant is subjected to successive distillation and extraction processes: (i) essential oil containing volatile phenols (e.g., carvacrol and eucalyptol) is removed with water steam, (ii) oil-free by-product is extracted with water to obtain the rosemary water extract (RWE), which is rich in hydrophilic polyphenols (e.g., rosmarinic acid) and (iii) the resulting by-product is extracted again with an acetone and water mix to obtain the rosemary lipophilic extract (RLE) [11,20,21]. RLE has been authorised by the European Union (E392) as an antioxidant for fish products at maximum doses of 150 mg/kg (carnosic acid + carnosol) (European Union Regulation 1129/2011/EU) [22]. The antioxidant effectiveness of different rosemary extracts has been checked in food products such as chicken and beef burgers [11,23], pork and chicken liver pâté [5,24,25], frozen salmon [18], fish patties [16], sea bream [17] and sardines [26]. In general, rosemary extracts showed good antioxidant properties in muscle-based products, although their use has some technological limitations. Rosemary extracts may not reach a suitable balance between dosage, polyphenols stability, preservative effects and sensory impact [27]. This might be due to various reasons: (i) polyphenol content of rosemary extract may vary depending on the raw material, which is related to genetic and agronomical variations, and the extraction procedure used; (ii) once the extract is added, rosemary polyphenols may degrade to a greater or lesser degree during food processing, particularly during thermal treatments and further storage; and (iii) rosemary extracts provide an intense herbal flavour that may negatively affect the acceptance of food products. Therefore, it is recommended to use typified rosemary extracts obtained from selected plants to attempt to standardise their preservative, sensory and nutritional properties [19].

Consumers increasingly demand healthier food products. Salmon pâté, a fatty product with a high caloric value, is often prepared with vegetable oils (e.g., sunflower oil) that provides a spreadable texture and a good flavour to the product. In less-caloric formulations, vegetable oils can be partially replaced by linseed [28], an ingredient with a neutral flavour and good thickening properties that can be used as a fat substitute and emulsion stabiliser, while also being a good source of n-6 PUFA and dietary fibre [29,30]. The working hypothesis of the present study was that RLE, applied at authorised doses, can stabilise lipids against oxidation in a PUFA-enriched fish pâté. The objective was therefore to test RLE (rich in carnosic acid and carnosol and obtained from selected plants) as a lipid antioxidant in a pasteurised-chilled salmon pâté made with linseed.

## 2. Materials and Methods

### 2.1. Experimental Design

A randomised statistical design was performed for the experiment. Three doses of RLE were tested in a PUFA-enriched salmon pâté formulated with linseed: (i) Low (R21): 0.21 g/kg, (ii) Medium (R42): 0.42 g/kg and (iii) High (R63): 0.63 g/kg. R63 treatment provided 150 mg carnosic acid + carnosol per kg pâté, the maximum dose authorised by EC [22]. Lipid oxidation (primary and secondary) and some related parameters (colour and pH) were assessed in salmon pâté just after pasteurisation and further displayed for 42 days. Sample size was *n* = 9 (3 pâté jars × 3 fabrication batches). A two-way ANOVA (Repeated Measures Design) was performed to determine the effects of treatments (RLE and storage time) on the dependent variables. The Tukey’s range test was used. Data were analysed with the Statistix 8.0 software for Windows (Analytical Software, Tallahassee, FL, USA).

### 2.2. Obtention of Rosemary Lipophilic Extract (RLE)

Selected high-yielding rosemary plants from a germplasm bank were cultivated and harvested in the experimental farm of the Institute of Agricultural and Food Research and Development (IMIDA, Murcia, Spain). RLE was obtained from the leaf by-product that had been previously distilled with water steam and extracted with water to obtain the essential oil and the RWE, respectively [27]. Then, 3 g of rosemary by-product were mixed with 450 mL acetone:water (1:1, *v*/*v*) and kept under constant stirring (30 °C for 90 min). The mix was centrifuged in a Digecen 21 R centrifuge (Orto Alresa, Madrid, Spain) (4560× *g* and 5 °C for 10 min) and the supernatant was then filtered (Whatman No. 4). The acetone fraction was removed by applying a vacuum at 40 °C in a Syncore Polyvap R-96 evaporation system (Buchi, Flawil, Switzerland), and the water fraction was removed using a lyophilisation system (Lyobeta 15, Telstar, Terrassa, Spain) (100 mbar and −80 °C for 24 h). Lyophilized RLE, a yellow-greenish dry powder with an intense herbal flavour, was stored at −80 °C until further use.

### 2.3. Determination of Rosemay Polyphenols

Polyphenols were determined in both RLE and treated pâté (carnosic acid and carnosol) according to Jordán et al. (2013) [20]. For RLE, 0.03 g dry extract was reconstituted with 5 mL methanol and filtered (0.22 µm nylon filter). For pâté, a 1 g sample was homogenised (ice covered) with 75 mL methanol at 10,000 rpm for 1 min in a Silent Crusher homogeniser (Heidolph, Schwabach, Germany), sonicated (ice-covered for 30 min) in a KM-UCI-150 system (R Espinar S.L., Barcelona, Spain) and centrifuged at 3900× *g* and 5 °C for 10 min). Methanol was evaporated under vacuum at 40 °C and the resulting dried extract was reconstituted again with methanol to 2 mL. Next, 20 µL of the methanolic sample solution (RE and pâté) were injected into an HPLC-1200 Series (Agilent, Waldbronn, Germany) equipped with a G1311A binary pump and a G1315A UV/Vis PDA detector. A Zorbax SB-C18 reversed-phase column (4.6 × 250 mm × 0.25 µm), with a Zorbax SB-C18 precolumn (4.6 × 125 mm × 0.25 µm) (Agilent Technologies, Santa Clara, CA, USA), was used. The flow rate was 1.0 mL/min and the mobile phases were acidified (0.05% formic acid) water (A) and acetonitrile (B). The elution gradient was: (i) min 0: 95% A/5% B; (ii) min 10: 85% A/15% B; (iii) min 30: 75% A/25% B; (iv) min 35: 70% A/30% B; (v) min 50: 45% A/55% B; (vi) min 55: 10% A/90% B; and (vii) min 70: 100% B, maintained for 10 min before returning to the initial conditions. Identification of polyphenols was performed by comparing retention times and spectra of different commercial standards. Detection wavelengths were 280 and 330 nm. Quantification was carried out using linear regression models based on standard dilution techniques. The method precision was determined according to the International Conference on Harmonisation guidelines [31]. Results were expressed as mg/g.

### 2.4. Salmon Pâté Manufacturing

Salmon pâté ingredients are shown in Table 1. Norwegian fresh salmon (*Salmo salar*) loins were purchased from Nordlaks Produkter AS (Murcia, Spain) and stored at −20 °C for 48 h. Thawed loins (at 4 °C/24 h) without skin and bones were cut in cubes (2 cm^3^) using a knife and cooked in water (2:1; *w*:*v*) at 90 °C for 10 min under stirring in a cooking robot (Taurus Mycook 1.6, Lérida, Spain). Before homogenising, cooked salmon cubes were minced for 10 min, skimmed milk was dissolved in water at 50 °C and linseed was hydrated at 1:2 (*w*:*v*) in a Silent Crusher homogeniser at 9500 rpm for 1 min. All ingredients (including flavouring, thickening, dye and RLE) were homogenised together for 10 min. Once paste was obtained, sterile glass jars (33-mL capacity) were manually filled and pasteurised in a water bath at 70 °C for 30 min. The jars were cooled in a water bath at 5 °C for 30 min and then kept for up to 42 days in a refrigerated display cabinet (Mod. 103899, Difri, Xirivella, Valencia, Spain) at 4 °C, 30% RH and white continuous illumination (600 lux). The antimicrobial efficiency of these pasteurisation conditions was checked in a previous trial [28]. Samples were stored at −80 °C before analyses.

### 2.5. Proximate Composition

Crude protein content was determined by the Kjeldahl method (reference 955.04; AOAC, 2000) [32]. Crude ash content was estimated by incineration (at 550 °C/24 h) in a furnace (reference 923.03; AOAC, 2000) [32]. Moisture content was determined with an infrared thermobalance (Model MA 50.R; Radwag, Radom, Poland). Available carbohydrates were estimated by weight difference. The energy value was calculated using standard conversion factors: 4 kcal/g for protein and carbohydrate and 9 kcal/g for fat, respectively [33].

### 2.6. pH and CIELab Colour

pH was determined with a MicropH 2001 pH meter (Crison, Barcelona, Spain) and a Cat. 52-22 combined electrode (Ingold Electrodes, Wilmington, DE, USA). CIELab colour was measured using a CR-200/08 Chroma Meter II (Minolta Ltd., Milton Keynes, UK) with a D65 illuminant, 2° observer angle and 50-mm aperture size. The results were expressed as CIELab values: lightness (L*), redness (a*) and yellowness (b*).

### 2.7. Lipid Extraction for Analyses

Pâté fat was extracted by the Folch method with some modifications [34]. First, 15 g of sample were mixed with chloroform:methanol (2:1, *v*/*v*) and 0.003% (*w*/*v*) BHT and then mixed with 1 M KCl. The organic phase was separated and dried in a Hei-vap rotavapor (Heidolph, Schwabach, Germany) at 30 °C and 337–470 mm Hg pressure. Fat content was gravimetrically determined (total lipids) and stored at −80 °C until analyses.

### 2.8. Peroxide Value (PV)

PV indicated the amount of hydroperoxides of fat pâté (AOCS Cd 8-53) [35]. In brief, 0.5 g fat was homogenised for 1 min with 10 mL chloroform, 15 mL of glacial acetic acid and 1 mL saturated potassium iodide water solution. The sample was kept in darkness for 5 min, mixed with 75 mL water and titrated with 0.002 N sodium thiosulphate solution in an automatic Titrino equipped with a 0160451100 PT WOC combined electrode (Metrohm Hispania, Madrid, Spain). Results were expressed as meq O_2_/kg fat.

### 2.9. Conjugated Dienes (CD) and Trienes (CT)

CD and TC were determined according to Pegg (2001) [36], with some modifications. A 0.1 g sample was dissolved in 10 mL isooctane and absorbance was measured at 233 nm (CD) and 268 nm (TC) in a UV/Vis spectrophotometer (Spectronic Unicam, New York, NY, USA). Results were expressed as µmol/g.

### 2.10. Thiobarbituric Acid Reactive Substances (TBARS)

TBARS were measured according to Botsoglou et al. (1994) [37] and calculated with a calibration curve (y = 1299.8x + 0.0033; R² = 0.999) of malonaldehyde (MDA) prepared with 1,1,3,3-tetraethoxypropane. Absorbance was measured at 532 nm (Unicam Spectrophotometer). Results were expressed as mg MDA/kg.

### 2.11. Fatty Acids (FA)

FA were identified and quantified as described by Cardenia et al. (2015) [34]. Twenty mg of lipid extract were treated with 200 μL of diazomethane before adding undecanoate methyl ester (CAS 1731-86-8, Sigma-Aldrich, St. Louis, MO, USA) (internal standard, 1 mg/mL) and transmethylating with 40 μL of 2 N KOH in methanol. A 6890N series gas chromatograph (Agilent Technologies, Madrid, Spain) equipped with a flame ionization detector (GC-FID) and an Agilent HP88 capillary column (60 m × 250 µm × 0.20 µm) was used for analysis. The operating conditions were: (i) injection volume: 1 mL; (ii) flow rate: 1.4 mL He/min and a 1:1 split; (iii) injector/detector temperatures: 250/260 °C; (iv) oven temperature program: 125 °C to 145 °C at 8 °C/min; hold at 145 °C for 26 min; from 145 °C to 220 °C at 2 °C/min; hold at 220 °C for 1 min. A fatty acid methyl esters (FAME) Mix C4–C24 (Supelco, Bellefonte, PA, USA) was used as standard. Results were expressed as g FAME /100 g fat.

### 2.12. Sterols and Cholesterol Oxidation Products (COP)

Sterols were extracted and purified according to Cardenia et al. (2015) [34]. About 200 mg of lipid extract containing internal standards for quantification of cholesterol and COP (141.12 µg of betulinol (Sigma Chemical, St. Louis, MO, USA) and 13.38 µg of 19-hydroxycholesterol (Steraloids, Newport, RI, USA), respectively) were cold saponified. Then, 100 µL of the unsaponifiable fraction were used for determining sterol composition, while the rest (900 µL) was subjected to SPE-NH_2_ for COP purification. Both sterol and COP fractions were derivatized with 1 mL of a silylating mixture (pyridine:hexamethyldisilazane:trimethylchlorosilane, 5:2:1, *v*/*v*/*v*), left standing at 40 °C for 20 min, dried under a nitrogen stream, re-dissolved in *n*-hexane (300 µL and 20 µL for sterols and COP, respectively) and injected into a Fast gas-chromatograph/mass-spectrometer (Fast GC/MS) as reported by Cardenia et al. (2012) [38], with slight modifications. For COP analysis, the split ratio was 1:15. Mass spectra and retention times of chemical standards (Sigma Chemical; Steraloids (Newport, RI, USA); Avanti Polar Lipids (Alabaster, AL, USA)) were compared with the chromatographic peaks of fish pâté for correct identification of sterols and COP. Both sterols and COP were quantified in the SIM acquisition mode, using calibration curves for each chemical compound, and their results were expressed as mg/100 g of pâté and µg/100 g of pâté, respectively. The proportion of total cholesterol oxidation (OR) was calculated as follows: OR = [(Total COP/Total cholesterol) × 100]/1000 [34]. Nine independent replicates were made for each sample.

### 2.13. Volatile Organic Compounds (VOC)

VOC were determined by headspace solid phase-micro-extraction (HS-SPME) according to Ortuño et al. (2021) [39], with some modifications. First, 5 g pâté were placed in amber 20-mL screw-capped vials (Agilent Technologies, Frankfurt, Germany) and flushed with nitrogen (at 275 kPa for 5 s). Before HS-SPME, the vials were kept in a water bath (at 40 °C for 10 min). VOC extraction was carried out in a water bath MPS 2XL autosampler (at 40 °C for 45 min) with continuous shaking (250 rpm) (Gerstel, Mülheim an der Ruhr, Germany). Analyses were performed with a 7890B Agilent gas chromatograph coupled to a 5977 A MSD mass spectrometer (GC-MS) using a VF-WAXms capillary column (30 m × 0.25 mm i.d. × 0.5 μm f.t.). Operating conditions were: needle immersion depth: 2.5 cm; vaporisation chamber diameter: 0.75 mm; desorption time inside the injector: 5 min; injection port and ionisation source temperature: 250 and 280 °C, respectively. The oven program was: 40 °C to 150 °C at 2.5 °C/min and then taken to 250 °C at 10 °C/min. Identification of individual compounds was performed by comparison of the spectra with the NIST 98 mass spectrometry library (NIST, Gaithersburg, MDN) and information obtained in previous trials [39]. Results (relative abundance) were expressed as Arbitrary Units (AU) × 10^6^.

## 3. Results

**Figure 1:**
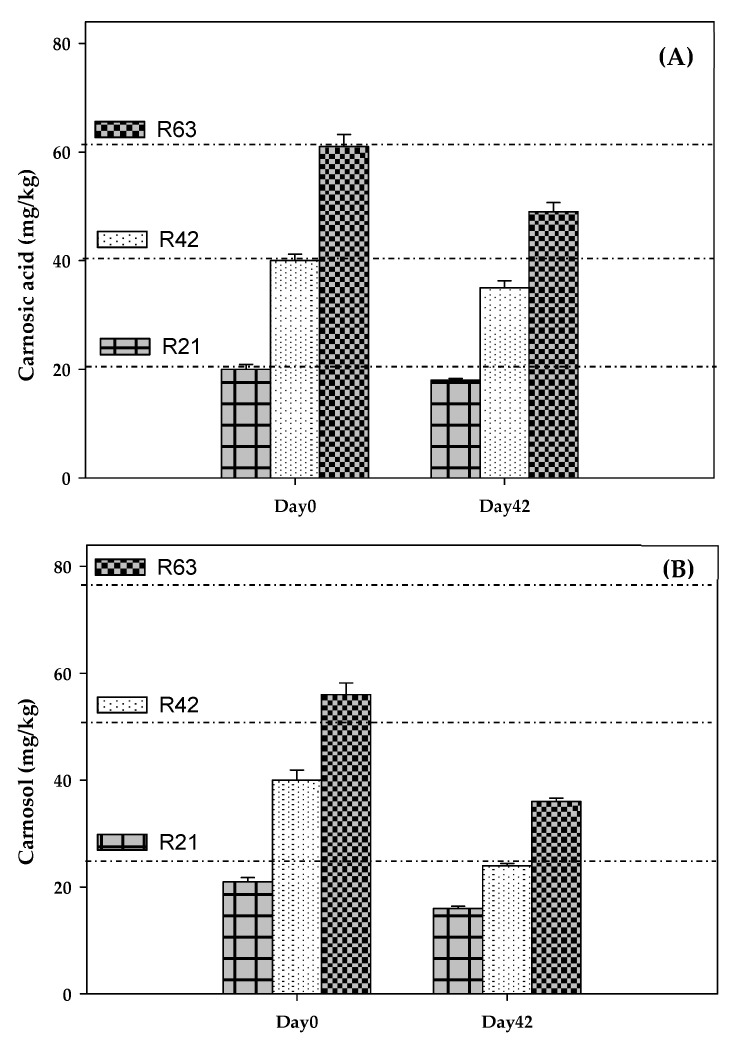
Carnosic acid (**A**) and carnosol (**B**) content (mg/kg) of salmon pâté. Abbreviations: R21/42/63: rosemary lipophilic extract at different doses.

Twenty-two polyphenols were determined in RLE (Table 2), including phenolic acids (salvianic, caffeic, isoferulic, rosmarinic, salvianolic), flavonoids (lithospermic acid, luteolin-4-glucoside, cirsimaritin, genkwanin, hesperidin, salvigenin and protocateic acid) and diterpenes (carnosol, carnosic acid, 7-methyl-rosmanol, 12-methyl-carnosic acid, rosmanol and different derivatives from carnosol and methyl-rosmanol). Carnosol and carnosic acid were the most abundant compounds (125 and 98 mg/g, respectively), representing 67% of total polyphenols in RLE (334 mg/g). Degradation of carnosic acid and carnosol was studied in salmon pâté (Figure 1). According to the polyphenol content of RLE, the initial quantities (mg/kg) of carnosic acid + carnosol added to raw pâté were: 21 + 26 (R21), 41 + 53 (R42) and 62 + 79 (R63). RLE also contained relevant levels of methyl-carnosic acid, carnosol derivatives and other diterpenes. On day 0, the concentrations (mg/kg) of carnosic acid + carnosol determined in pâté were: 20 + 21 (R21), 40 + 39 (R42) and 61 + 56 (R63), while, on day 42, these concentrations were: 18 + 16 (R21), 35 + 24 (R42) and 49 + 35 (R63). Thus, both diterpenes were retained without degrading in the pasteurised salmon pâté, even though a part (10–18% of carnosic acid and 24–38% of carnosol) degraded during chill storage.

Salmon pâté contained 12 g/100 g total protein, 19 g/100 g total lipids and 61 g/100 g moisture, contributing with 270.6 kcal/100 g (Table 3). Data regarding general lipid oxidation, CIELab colour and pH are shown in Table 4. RLE addition did not affect PV on day 0, but R63 pâté presented the highest PV on day 42, while not impacting CD and CT values on day 0 and 42. Thus, PV discriminated the effects of RLE on primary lipid oxidation better than CD and CT, which provide similar information. Chill storage did not affect PV but actually decreased CD and CT values. The antioxidant effects of RLE addition on lipids were confirmed by TBARS. MDA levels were higher in the untreated pâté on day 0 and 42 than in the pâté with RLE (at any dose). No differences were found in the MDA levels among R21, R42 and R63 pâtés. Similarly, adding RLE did not affect colour on day 0, while pâtés with more RLE (R42 and R63) had lower values of L* and a* and higher values of b* and h* than the untreated pâté on day 42. Therefore, salmon pâté underwent some discolouration (slight browning) during chill storage that might be inhibited using RLE. Regardless of the dose used, RLE slightly decreased the pâté pH on days 0 and 42. Overall, the pH values hardly decreased by around 0.2 after chill storage.

Twenty-two FA were quantified (g FA/100 g fat) (Table 5). The most abundant class was MUFA (22.7–26.2 g/100 g fat) with C18:1*t* n-9 as main FA (18.3–22.2 g/100 g fat), followed by PUFA (17.3–20.2 g/100 g fat), with C18:2*c* n-6 (11.8–13.8 g/100 g fat) and C18:3α n-3 (2.1–2.7 g/100 g fat) as major FA, and by SFA (9.2–10.6 g/100 g fat), with C16:0 as predominant FA (3.6–4.6 g/100 g fat). Levels of EPA and DHA were around 1 and 1.4 g/100 g fat, respectively. RLE addition did not affect the FA levels on day 0 and 42, while chill storage only led to some changes in minor FA. TFA content was similar for the untreated and the RLE pâtés on day 0 and 42. Consequently, nutritional FA ratios (n-6/n-3 and P/S) were unaffected by RLE addition or chill storage.

The most abundant sterol was cholesterol (33.7–41.7 mg/100 g pâté), followed by β-sitosterol (12.9–17.8 mg/100 g pâté), campesterol (2.3–3.9 mg/100 g pâté) and stigmasterol (1.6–2.2 mg/100 g pâté) (Table 6). Campesterol content was higher in the untreated than in the R21 and R63 pâtés on day 0, whereas on day 42 a reduction was observed for all sterols in all R21 pâtés with respect to untreated ones. Chill storage generally led to a decrease in sterol content, except for stigmasterol. Among the sterol oxidation products, only COP were determined, as cholesterol was the most abundant sterol in salmon pâté. The main COP was 7-ketocholesterol (7-KC), followed by 7α-hydroxycholesterol (7α-HC), 7β-hydroxycholesterol (7β-HC), cholestane-3β,5α,6β-triol (triol), 5β,6β-epoxycholesterol (β-EC) and 5α,6α-epoxycholesterol (α-EC). RLE addition had an antioxidant effect on cholesterol, since the untreated pâté presented the highest COP contents on day 0 and 42. Consequently, the OR were clearly higher in untreated (0.49–0.70%) than in pâtés added with RLE (0.22–0.32%). As observed for single and total COP, RLE addition, storage time and its interaction influenced this ratio. OR increased with chill storage in untreated and R21, while remaining stable in R42 and R63 pâtés.

A total of twenty VOC were identified in the headspace of salmon pâté including several hydrocarbons (3-methoxy-1-propene, toluene, butylated-hydroxytoluene, 1-ethyl-4-methyl-benzene, and 1H-trindene, 2,3,4,5,6,7,8,9-octoctahydro-1,1,4,4,9,9-hexamethyl), alcohols (1-pentanol, 1-hexanol, 1-penten-3-ol, (Z)-2-penten-1-ol, 1-octen-3-ol, eugenol, and benzyl alcohol), aldehydes (hexanal, 2-methyl-propanal, 2-(phenylmethylene)-octanal and 2,5-bis(trimethyl)-benzaldehyde), ketones (2’,6’-dihydroxyacetophenone), organic acids (2-amino-4-methyl-benzoic acids) and terpenes (D-limonene and α-pinene). The relative abundance of most of these VOC was unaffected by the addition of RLE or chill storage, with some exceptions (Table 7). Of these, 1-pentanol was less abundant in the untreated than in the pâté with RLE (at any dose) on day 42. Overall, the abundance of 1-pentanol and 1-hexanol increased with chill storage, while, in contrast, the abundance of hexanal and 2-methyl-propanal decreased on day 42. The above four VOC were studied as possible lipid oxidation markers in salmon pâté.

## 4. Discussion

Recovery of rosemary polyphenols depends on the raw materials, solvents and operating conditions applied to obtain the extracts [19,40]. Different studies agree that carnosic acid and carnosol are the most abundant polyphenols present in RLE [19,21,40]. The reported concentration ranges are 47–179 mg/g for carnosic acid, 5–28 mg/g for carnosol and 50–200 mg/g for total polyphenols [19,41]. Thus, the RLE tested in the present study was particularly rich in polyphenols (333 mg/g), which was one of the main objectives of the plant’s selection. As a purity criterion, European Union Directive 2010/67/UE [42] establishes that the RLE for food application must contain at least 10% (*w*:*w*) of carnosic acid plus carnosol, a percentage widely exceeded by the RLE used in this experiment. Assessment of the stability of carnosic acid and carnosol provides an idea of how rosemary antioxidants behave in food matrices. Under oxidising conditions, carnosic acid is transformed into carnosol [21,43], which can be regenerated or not by other antioxidants acting in the food matrix. As observed in the experimental data, both diterpenes were quite resistant to the pasteurisation conditions applied, as has also been reported for carnosic acid in pork liver pâté [24]. In general, rosemary diterpenes are quite resistant to the cooking procedures applied in food [24,41]. In the present study, the degradation of carnosic acid and carnosol mainly occurred during chill storage. This was expected, since salmon pâté was aerobically homogenised, which favours the presence of occluded oxygen, and jars were kept under refrigeration and fluorescent lighting (600 lux) for 42 days. Doolaege et al. (2012) [24] found that a part (6–32 mg/kg) of the added carnosic acid (250–750 mg/kg) degraded in liver pâté kept at 4 °C for 48 h. There were no available data on the stability of rosemary polyphenols in other studies on the antioxidant properties of RE in meat and fish products [11,23].

The formulation used for salmon pâté in the present study enabled the reduction of fat content and caloric intake compared to commercial fish pâtés (30% fat and 330 kcal/100 g pâté) [44,45,46]. Salmon pâté reflected the FA and sterol profiles of the different fat sources used as ingredients (salmon muscle, sunflower oil and linseed). The FA profile was similar to those reported for other fish pâtés [45,46,47]. The relevance of the different FA classes (MUFA > PUFA > SFA) is coherent with the above-mentioned fat sources, since salmon flesh and linseed are rich in MUFA and PUFA [30,48], while sunflower oil is rich in PUFA [49]. Salmon muscle is rich in cholesterol, while β-sitosterol is the main phytosterol in sunflower oil and linseed [50,51]. Sterol content tended to decrease in all pâtés following chilled storage, likely due to the oxidation reactions involving lipids. The cholesterol content of the salmon pâté coincides with that reported by Echarte et al. (2004) [47]. From a nutritional standpoint, salmon pâté showed a n-6/n-3 ratio (2.3–2.6) below the recommended level of 4 [52], resulting in a balanced dietary source of n-3 FA. An adequate n-6/n-3 ratio in the diet contributes to optimising bioavailability, metabolism and incorporation of FA into membrane phospholipids [30]. Likewise, the P/S ratio (1.79–1.97) was above the minimum recommended (0.5–0.7) [52], which is considered a good nutritional trait. An increase in the P/S ratio can lead to reduced plasma total cholesterol [52], while, at the technological level, it is a useful indicator of fat oxidation susceptibility, which mainly affects PUFA. In the present study, pâté lipids, integrated by a high proportion of PUFA, were quite stable during chill storage, perhaps partly due to linseed, a thickening agent containing hydrocolloids that might help stabilising the emulsion [53].

Rosemary polyphenols, containing benzene rings able to act as free radical scavengers, fulfilled their antioxidant role delaying lipid oxidation in the pâté; however, in quantitative terms, the addition of up to 210 mg/kg of rosemary polyphenols does not seem enough to protect a fatty fish emulsion (19 g fat/100 g pâté, of which, 4.7 g MUFA and 3.4 g PUFA) against oxidation. Lipid oxidation reactions involve three stages: initiation, propagation and termination. Some techniques measure the loss of initial reactants (such as oxygen, lipid and antioxidants), others the formation of primary oxidation products (such as hydroperoxides and conjugated dienes) and others the formation of secondary oxidation products (such as alcohols, aldehydes, hydrocarbons and ketones) [54]. Thermal exposure in the presence of oxygen induces lipid oxidation, which forms hydroperoxides and causes double bond displacement or isomerisation, resulting in an increased production of CD and CT from unsaturated FA [55]. As oxidation advances in pâté during the display period, secondary oxidised lipids are formed to the detriment of the early oxidised lipids, even though they may still be generated. This may explain why primary oxidation stabilised or decreased after chill storage, as indicated by the reduction observed in the CD and CT values. The reactive chemical species that gave rise to CT evolved towards other secondary compounds such as TBARS [56]. PV was the sole index that discriminated the antioxidant activity of rosemary polyphenols in the early stages. As observed in Table 4, R63 pâté clearly had more hydroperoxides than the other samples on day 42, suggesting that the formation of secondary oxidised lipids such as TBARS and VOC was inhibited. Rosemary polyphenols may inhibit the formation of oxidised lipids through hydrogen donation or preventing the cleavage of lipid hydroperoxides [55].

TBARS is a widely used index with technological (flavour and rancidity) and nutritional (toxicity) implications that measures the levels of aldehydes and other secondary oxidised lipids in the pâté. Lipid oxidation is enhanced by the thermal treatment and favoured by fat unsaturation and the presence of oxygen. Salmon pâté presented an incipient lipid oxidation just after pasteurisation. The antioxidant role of RLE during pâté preparation was modest, since it only slightly inhibited the formation of MDA and was not dose-dependent. Salmon was cooked before adding RLE, which would explain why there were no marked differences regarding MDA levels among the different freshly pasteurised pâtés. TBARS formation continued during further retail display. On day 42, MDA level practically doubled in the pâté formulated without RLE, reaching TBARS values near 2 mg MDA/kg, the threshold value from which rancidity is sensorially detected in cooked meat [57]. In contrast, RLE clearly inhibited MDA formation in the chill-stored pâté. However, increasing RLE dose did not improve results. In other studies, lipid oxidation was inhibited when using different RLE in pork liver pâté [24,58], chicken pâté [5] or RWE in frozen salmon [18] and refrigerated sardines [26], confirming the present findings.

The antioxidant effect of RLE was also confirmed for cholesterol. The COP profile found in this study is similar to that reported by Echarte et al. (2004) [47] for salmon pâté, where the most abundant COP originated from the monomolecular oxidation reaction pathway involving B ring (i.e., 7-oxysterols), while epoxy and triol derivatives, generated from a bimolecular reaction pathway, were found in smaller quantities (β-EC and triol) or even undetected (α-EC) in the pâtés with RLE. The amounts of total COP formed do not correspond to the observed decrease in cholesterol, which might be due to the reaction of COP with amino groups from amino acids, peptides and proteins leading to the formation of Schiff bases [59] and/or the formation of mid-polarity sterol oxidation products [60] that cannot be determined under the analytical conditions used. Total COP and OR showed the same trend as TBARS, with values in untreated samples that were about twice as much as those found in pâtés with RLE at 0 and 42 days of chilled storage. Cholesterol oxidation is known to be favoured in the presence of oxygen and during chilled display [61], but RLE addition was able to greatly hinder COP formation, without a dose-dependent effect.

RLE addition did not initially affect pâté colour, as also reported in pork liver pâté with RLE [25]. It is unlikely that a small amount of RE can alter the colour of an orange salmon pâté that was also coloured with capsanthin. Other studies on pork liver pâté made with tea and grape seed extracts [62] and with seaweed extracts [56] reported some changes in CIELab colour due to the contribution of pigments such as chlorophylls [63] or by the action of polyphenol oxidases able to condense quinones, forming dark compounds [62,64]. As these enzymes are inactivated by heating, it is unlikely that enzymes from a rosemary by-product (distilled with hot water steam) remain active in a thermally treated pâté. The untreated pâté showed some discolouration on day 42, as indicated by the increased Hue angle, a browning index, thus evidencing a general oxidation of product. Discolouration processes affecting the cooked–chilled muscle products may be ascribable to metmyoglobin formation, the denaturation of myofibrillar proteins producing colour changes when interacting with myoglobin [18,65] and lipid oxidation [25]. Similarly, colour could be stabilised in pork liver pâtés with RLE [24,25] or with beer residue, chestnut leaves and peanut skin [66], as well as in frozen salmon with rosemary whole extract [18].

The pH of pâté was slightly lower when RLE was incorporated into its formulation. Similar results were found in pork liver pâté made with RE [24] and with other vegetable extracts [62,66]. The natural acidity of plant extracts may decrease the pH of treated pâtés [62]. In the present study, this effect was not dose-dependent, which suggests that some components of RLE were implicated in chemical reactions involving small changes in pH. The pH dropped slightly more (by 0.2) after storage in all pâtés, formulated with and without RLE, likely due to oxidation phenomena, since microbial events often result in more pronounced changes in pH and, in addition, the efficacy of pasteurisation treatment had been checked previously [28]. Moreover, carnosol and carnosic acid may present bacteriostatic activities, as these can alter the lipidic order of phospholipidic membranes [67].

A relevant part of the VOC identified in headspace corresponded to C5–C8 aliphatic aldehydes and alcohols. Hexanal has been identified as the most abundant VOC in sunflower and linseed oil blends, together with other VOC (propanal, pentanal, 1-penten-3-one, 1-pentanol, octanal, 1-octen-3-one, 1-octen-3-ol and (E,Z)-2,4-heptadienal) that contribute to oil aroma [68]. In addition, saturated and unsaturated aliphatic VOC can be generated from the thermal degradation and oxidation of C18–C20 MUFA and C18–C22 PUFA present in fish lipids. In fact, propanal and hexanal are the most abundant VOC detected in cooked salmon aroma [69,70]. Benzene derivatives at different oxidation stages (e.g., toluene; benzaldehyde and benzoic acid) can also be formed in cooked salmon from aromatic precursors [69,70]. BHT can be used to stabilise carotenoid dyes, which might explain why BHT appeared in pâté headspace. α-Pinene and eugenol are volatile terpenes that can proceed from rosemary by-products [71] or can be directly formed in cooked fish [69,70]. Unlike the TBARS trend, the VOC levels, except for 1-pentanol, did not allow discrimination of the antioxidant effect of RLE on salmon pâté lipids. For instance, inhibition by RLE of hexanal, a well-known VOC generated by cooking, could not be proven. The VOC profile of salmon pâté suggests that vegetable VOC from sunflower and linseed predominated over VOC from fish in headspace under the conditions used for HS-SPME. The number of VOC identified in salmon pâté was actually modest compared to those reported for cooked salmon [69,70]. Moreover, many characteristic C9–C11 aliphatic aldehydes (e. g. nonanal and nonenal) of flesh origin were not detected. In general, the VOC detected in salmon pâté seems to provide little information about lipid oxidation and would thus be of little interest as lipid oxidation markers. In any case, sampling of different manufacturing batches might have increased intra-group variability in parameters (FA, sterols and others) related to the composition of pâté, making treatment effects less evident.

## 5. Conclusions

Food applications for rosemary extracts require standardising their polyphenol content. The crop of high-yielding selected rosemary plants can help in improving and standardising these extracts. As reported in the present study, the rosemary lipophilic extract used is rich in polyphenol antioxidants, particularly diterpenes, and provides good results in salmon pâté, a product prone to oxidation. Rosemary diterpenes resist pasteurisation without degrading and can act as antioxidants during the shelf-life of pasteurised pâté. As a result, lipids are stabilised against oxidation and salmon pâté contains less secondary oxidation compounds, in all likelihood being healthier. However, this did not entail relevant changes in the fatty acid content or relative abundance of volatile organic compounds from lipids. Therefore, oxidative deterioration of these types of fish emulsions can be naturally controlled with rosemary extracts rich in diterpenes, thus representing a valid alternative for the formulation of clean label seafood products. It is not necessary to reach the maximum quantity of rosemary diterpenes (150 mg/kg) authorized for fish products to obtain a suitable antioxidant effect in this PUFA-enriched pâté. Most of the oxidation indices improved in the salmon pâté when a third of the aforementioned dose was used.

## Figures and Tables

**Table 1:** Ingredients of salmon pâté.

	g/100 g
Salmon loin	50.00
Water	32.45
Sunflower oil	9.30
Linseed grain way	3.00
HP Inulin	0.20
Milk powder/sodium caseinate	4.00
Salt	1.00
Paprika orange dye	0.05
Rosemary lipophilic extract (RLE) *	≤0.063

* Added at 0.021 (R21), 0.042 (R42) and 0.063 g/100 g (R63); 150 mg/kg carnosic acid + carnosol).

**Table 2:** Polyphenol content (mg/g) of rosemary lipophilic extract.

	Mean	RMSE
Phenolic acids		
Salvianic acid	0.49	0.023
Caffeic acid	0.25	0.021
Isoferulic acid	0.22	0.033
Rosmarinic acid	9.16	0.161
Salvianolic acid	3.59	0.082
Flavonoids		
Lithospermic acid	2.93	0.041
Luteolin-4-glucoside	0.45	0.034
Cirsimaritin	7.06	0.163
Genkwanin	5.55	0.180
Genkwanin 4’-methyl ether	7.08	0.253
Hesperidin	5.16	0.132
Salvigenin	6.22	0.150
Protocateic acid	0.03	0.012
Diterpenes		
Carnosol	125.22	2.273
Carnosic acid	97.91	1.671
7-Methyl-rosmanol	13.49	1.083
12-Methyl carnosic acid	18.36	1.260
Carnosol derivative (1)	4.53	0.181
Rosmanol	13.57	1.022
Methyl Rosmanol derivative (1)	2.50	0.050
Methyl Rosmanol derivative (2)	4.55	0.082
Carnosol derivative (2)	5.20	0.041
Total polyphenols	333.50	

Abbreviations: RMSE: Root Mean Standard Error. Limit of Quantification (LoQ): 0.01 mg/g.

**Table 3:** Proximate composition (g/100 g) and caloric value (kcal/100 g) of salmon pâté.

	Mean	RMSE
Moisture	61.36	0.102
Total Protein	11.80	0.113
Total Lipids	19.10	0.171
Total carbohydrates	5.72	0.182
Ash	2.04	0.021
Caloric value	270.58	

Abbreviations: M: Mean; RMSE: Root Mean Standard Error.

**Table 4:** Lipid oxidation indexes, CIELab colour (CIELab units) and pH of salmon pâté.

		Untreated		R21		R42		R63			Effects (*p*-Values)		
	Day	Mean		Mean		Mean		Mean		RMSE	RLE	ST	RLE × ST
PV (meq O_2_/kg)	0	4.71		4.10		4.02		4.34		0.261	ns	ns	*
	42	3.92	^b^	3.82	^b^	4.06	^b^	5.37	^a^	0.281			
CD (µmol/g)	0	1.82		1.79		1.83		1.92		0.100	ns	***	ns
	42	1.46		1.55		1.55		1.46		0.111			
CT (µmol/g)	0	0.76		0.81		0.78		0.83		0.049	ns	***	ns
	42	0.62		0.52		0.64		0.58		0.055			
TBARS (mg MDA/kg)	0	0.86	^a^	0.79	^b^	0.79	^b^	0.79	^b^	0.033	***	***	***
	42	1.55	^a^	0.94	^b^	0.91	^b^	0.89	^b^	0.042			
L *	0	71.28		71.01		70.94		70.72		0.285	ns	ns	ns
	42	72.12	^a^	71.00	^b^	70.82	^b^	70.65	^b^	0.174			
a *	0	14.22		14.35		13.98		13.87		0.387	ns	***	ns
	42	14.68	^b^	15.51	^ab^	15.91	^a^	15.98	^a^	0.352			
b *	0	40.23		40.36		40.32		40.31		0.588	ns	ns	ns
	42	39.30	^b^	40.47	^ab^	40.63	^a^	40.60	^a^	0.441			
Hue angle	0	70.58		70.43		70.89		71.01		0.326	ns	***	ns
	42	69.51	^a^	69.05	^ab^	68.62	^b^	68.52	^b^	0.297			
pH	0	6.23	^a^	6.09	^b^	6.03	^b^	6.03	^b^	0.055	**	***	ns
	42	6.05	^a^	5.80	^b^	5.83	^b^	5.77	^b^	0.069			

Abbreviations—R21/42/63: rosemary lipophilic extract at different doses; ST: storage time; RMSE: Root Mean Standard Error; TBARS: Thiobarbituric acid reactive substances; MDA: malondialdehyde; PV: Peroxide Value; CD: Conjugated dienes; CT: Conjugated trienes. ^a, b^ RLE addition effects at the same storage time (One-way ANOVA). *p*-values: overall effects of RLE and ST (Two-way ANOVA; Repeated Measures Design). Significance levels: *** *p* < 0.001; ** *p* < 0.01; * *p* < 0.05; ^ns^ *p* > 0.05.

**Table 5:** Fatty acid content (g FAME/100 g fat) and related nutritional indexes of salmon pâté.

		Untreated	R21	R42	R63		Effects (*p*-Values)		
	T	Mean	Mean	Mean	Mean	RMSE	RLE	ST	RLE × ST
C10:0	0	0.32	0.35	0.34	0.39	0.026	ns	ns	ns
	42	0.38	0.38	0.36	0.33	0.032			
C14:0	0	0.81	0.77	0.89	0.78	0.057	ns	ns	ns
	42	0.82	0.90	0.88	0.88	0.083			
C15:0	0	0.23	0.25	0.25	0.26	0.170	ns	**	ns
	42	0.29	0.30	0.28	0.26	0.015			
C16:0	0	4.18	3.97	4.55	3.94	0.327	ns	ns	ns
	42	4.13	4.17	4.45	4.60	0.477			
C16:1 n-7	0	0.79	0.74	0.86	0.74	0.058	ns	ns	ns
	42	0.79	0.86	0.85	0.86	0.090			
C17:0	0	0.27	0.29	0.30	0.30	0.020	ns	***	ns
	42	<0.01	<0.01	<0.01	<0.01				
C18:0	0	1.68	1.88	1.90	1.87	0.225	ns	ns	ns
	42	1.85	2.06	2.21	2.02	0.238			
C18:1*t* n-9	0	19.99	20.48	22.26	20.16	1.453	ns	ns	ns
	42	18.92	20.84	20.48	18.34	1.911			
C18:1*c* n-9	0	1.40	1.45	1.51	1.60	0.132	ns	ns	ns
	42	1.36	1.48	1.70	1.52	0.149			
C18:2 *c* n-6	0	12.40	11.82	12.94	11.93	0.990	ns	ns	ns
	42	12.02	12.18	13.16	13.80	1.616			
C20:0	0	0.64	0.69	0.70	0.71	0.045	ns	*	ns
	42	0.70	0.78	0.75	0.70	0.037			
C18:3α n-3 (ALA)	0	2.47	2.11	2.70	2.23	0.224	ns	ns	ns
	42	2.37	2.47	2.57	2.68	0.338			
C20:1 n-9	0	1.04	0.94	1.14	0.98	0.080	ns	ns	ns
	42	1.05	1.14	1.12	1.13	0.112			
C20:2 *c* n-6	0	0.49	0.47	0.53	0.49	0.033	ns	ns	ns
	42	0.52	0.56	0.54	0.53	0.036			
C20:3 n-6	0	0.22	0.23	0.24	0.24	0.015	ns	ns	ns
	42	0.23	0.26	0.25	0.24	0.012			
C20:3 n-3	0	0.41	0.44	0.46	0.48	0.029	ns	*	*
	42	0.48	0.51	0.47	0.45	0.024			
C22:0	0	0.53	0.53	0.58	0.56	0.034	ns	ns	ns
	42	0.58	0.62	0.60	0.57	0.300			
C22:1 n-9	0	0.39	0.40	0.43	0.42	0.027	ns	*	ns
	42	0.43	0.47	0.45	0.42	0.021			
C20:5 n-3 (EPA)	0	0.97	0.88	1.04	0.92	0.063	ns	ns	ns
	42	0.98	1.04	1.02	1.03	0.084			
C24:0	0	0.81	0.93	0.79	0.94	0.071	ns	ns	ns
	42	0.85	0.93	0.81	0.77	0.077			
C24:1	0	0.39	0.40	0.42	0.42	0.028	ns	*	ns
	42	0.42	0.46	0.44	0.41	0.021			
C22:6 n-3 (DHA)	0	1.42	1.31	1.50	1.36	0.081	ns	ns	ns
	42	1.43	1.49	1.46	1.47	0.095			
ƩTFA	0	51.88	51.37	56.34	51.75	3.027	ns	ns	ns
	42	50.59	53.90	54.83	53.05	4.769			
ƩSFA	0	9.50	9.68	10.30	9.76	0.593	ns	ns	ns
	42	9.59	10.14	10.32	10.14	0.683			
ƩMUFA	0	24.01	24.42	26.62	24.33	1.561	ns	ns	ns
	42	22.98	25.25	25.03	22.70	2.203			
ƩPUFA	0	18.38	17.27	19.41	17.66	1.333	ns	ns	ns
	42	18.02	18.51	19.53	20.21	2.171			
ƩHP-PUFA	0	5.50	4.98	5.95	5.24	0.387	ns	ns	ns
	42	5.48	5.78	5.77	5.87	0.535			
n-6/n-3 ^1^	0	2.47	2.71	2.40	2.54	0.161	ns	ns	ns
	42	2.40	2.34	2.39	2.56	0.098			
P/S ^2^	0	1.92	1.79	1.89	1.81	0.083	ns	ns	ns
	42	1.86	1.80	1.86	1.97	0.114			

Abbreviations—FAME: Fatty Acid Methyl Esters; ST: storage time; R21/42/63: rosemary lipophilic extract at different doses; RMSE: Root Mean Standard Error; FA: fatty acid; SFA: Saturated FA; MUFA: Monounsaturated FA; PUFA: Polyunsaturated FA. *c*: *cis*; *t*: *trans*; ALA: α-linolenic acid; LA: linoleic acid; EPA: eicosapentaenoic acid; DHA: docosahexaenoic acid. ∑ SFA = Sum of C10:0, C14:0, C15:0, C16:0, C17:0, C18:0, C20:0, C22:0 and C24:0. ∑ MUFA = Sum of *cis* and *trans* isomers of C16:1, C18:1, C20:1, C22:1 and C24:1. ∑ PUFA = Sum of *cis* and *trans* isomers of C18:2, C18:3, C20:2, C20:3, C20:5 and C22:6. ∑HP-PUFA: Sum of PUFA with three or more unsaturated bonds. ^1^ n-6/n-3 = Ʃn-6 PUFA/Ʃ n-3 PUFA. ^2^ $\frac{P}{S}$ (P/S Index) = $\frac{ƩPUFA}{ƩSFA}$. *p*-values: overall effects of RE and ST (Two-way ANOVA; Repeated Measures Design). Significance levels: *** *p* < 0.001; ** *p* < 0.01; * *p* < 0.05; ^ns^ *p* > 0.05. Limit of Quantification (LoQ): 0.01 g FAME/100 g fat.

**Table 6:** Phytosterols (mg/100 g), cholesterol (mg/100 g), COP (µg/100 g) and cholesterol oxidation ratio (%) of salmon pâté.

		Untreated		R21		R42		R63			Effects (*p*-Values)		
	Day	Mean		Mean		Mean		Mean		RMSE	RLE	ST	RLE × T
Campesterol	0	3.91	^a^	3.25	^b^	3.45	^ab^	3.09	^b^	0.230	**	***	ns
	42	3.59	^a^	2.33	^b^	2.73	^b^	2.57	^b^	0.256			
Stigmasterol	0	2.16		1.91		2.00		1.86		0.109	**	ns	ns
	42	2.19	^a^	1.64	^b^	1.89	^ab^	1.75	^b^	0.125			
β-Sitosterol	0	17.77		15.84		16.39		15.74		0.541	ns	*	ns
	42	17.42	^a^	12.85	^b^	14.88	^ab^	14.58	^ab^	0.886			
Cholesterol	0	40.58		39.97		41.65		40.00		1.651	ns	*	ns
	42	41.42	^a^	33.73	^b^	36.99	^ab^	37.23	^ab^	1.541			
7α-HC	0	51.38	^a^	18.13	^b^	14.10	^b^	18.23	^b^	2.695	***	*	ns
	42	54.00	^a^	20.49	^b^	16.24	^b^	21.57	^b^	3.501			
7β-HC	0	42.39	^a^	24.52	^b^	25.24	^b^	27.21	^b^	2.636	***	***	***
	42	65.32	^a^	26.96	^b^	26.55	^b^	29.62	^b^	2.623			
α-EC	0	13.91	^a^	<LoQ	^b^	<LoQ	^b^	<LoQ	^b^	0.317	***	***	***
	42	24.23	^a^	<LoQ	^b^	<LoQ	^b^	<LoQ	^b^	0.657			
β-EC	0	11.65	^a^	6.13	^b^	5.51	^b^	6.16	^b^	1.135	***	***	***
	42	21.42	^a^	7.26	^b^	6.53	^b^	6.45	^b^	1.457			
Triol	0	26.21	^a^	16.59	^b^	16.09	^b^	17.34	^b^	2.092	***	***	***
	42	45.61	^a^	18.33	^b^	17.85	^b^	18.60	^b^	2.681			
7-KC	0	51.79	^a^	31.08	^b^	32.48	^b^	26.98	^b^	2.093	***	***	***
	42	75.48	^a^	33.02	^b^	34.40	^b^	29.27	^b^	2.433			
Total COP	0	197.33	^a^	96.44	^b^	93.43	^b^	95.93	^b^	4.517	***	***	***
	42	286.06	^a^	106.07	^b^	101.56	^b^	105.51	^b^	4.271			
OR	0	0.49	^a^	0.24	^b^	0.22	^b^	0.24	^b^	0.016	***	***	**
	42	0.70	^a^	0.32	^b^	0.28	^b^	0.29	^b^	0.020			

Abbreviations—ST: storage time; L/M/H RLE: low/medium/high rosemary lipophilic extract; M: Mean; RMSE: Root Mean Standard Error; 7α-HC, 7α-hydroxycholesterol; 7β-HC, 7β-hydroxycholesterol; α-EC, 5α,6α-epoxycholesterol; β-EC, 5β,6β-epoxycholesterol; Triol, cholestane-3β,5α,6β-triol; 7-KC, 7-ketocholesterol; COP: cholesterol oxidation products; OR: cholesterol oxidation ratio. ^a, b^ RLE addition effects at the same storage time (One-way ANOVA). *p*-values: overall effects of RLE and ST (Two-way ANOVA; Repeated Measures Design). Significance levels: *** *p* < 0.001; ** *p* < 0.01; * *p* < 0.05; ^ns^ *p* > 0.05. Limit of Quantification (LoQ): 0.25 µg/100 g pâté.

**Table 7:** Relative abundance (AU × 10^6^) of four volatile organic compounds selected as possible lipid oxidation markers in salmon pâté.

		Untreated		R21		R42		R63			Effects (*p*-Values)		
	Day	Mean		Mean		Mean		Mean		RMSE	RLE	ST	RLE × ST
Hexanal	0	3.31		2.52		2.58		2.00		0.636	ns	***	ns
	42	0.49		0.27		0.29		0.10		0.072			
2-Methyl-propanal	0	35.30		34.10		31.80		29.00		3.428	ns	**	ns
	42	29.10		26.40		28.80		27.40		2.976			
1-Pentanol	0	0.27		0.83		0.95		0.85		0.254	***	***	ns
	42	1.19	^b^	2.38	^a^	3.13	^a^	3.00	^a^	0.338			
1-Hexanol	0	10.10		9.84		11.80		10.90		1.519	ns	***	ns
	42	13.80		12.00		12.80		13.70		1.593			

Abbreviations—R21/42/63: rosemary lipophilic extract at different doses; ST: Storage Time; M: Mean; RMSE: Root Mean Standard Error; AU: Arbitrary units. ^a, b^ RLE addition effects at the same storage time (One-way ANOVA). *p*-values: overall effects of RLE and ST (Two-way ANOVA; Repeated Measures Design). Significance levels: *** *p* < 0.001; ** *p* < 0.01; ^ns^ *p* > 0.05.

## Data Availability

Data is contained within the article.
